# Inhibition of Gastric Acid Secretion by Unfractionated and Low Molecular Weight Heparins in the Rat

**DOI:** 10.1100/tsw.2006.42

**Published:** 2006-02-22

**Authors:** Omar M.E. Abdel Salam, Ayman R. Baiuomy, Amany Ameen

**Affiliations:** Department of Pharmacology, National Research Centre, Tahrir St., Dokki, Cairo, Egypt

**Keywords:** unfractionated heparin, low molecular weight heparins, gastric acid secretion, histamine, bethanechol, urethane-anesthetized rat

## Abstract

The majority of patients receiving heparin preparations are at stress, which is a risk factor for the development of gastric erosions. Our aim was to examine the effect of unfractionated heparin (UFH) and low molecular weight heparins (LMWHs) on gastric acid secretion. Gastric acid secretion was induced in urethane-anesthetized rats by distention of the stomach (2 ml saline for 2 h) in addition to histamine or bethanechol stimulation. Distension-stimulated acid secretion (2 ml for 2 h) was significantly inhibited by intraperitoneal administration of UFH (2000 IU/kg, 19% reduction), enoxaparin (180 or 360 IU/kg, 59.2 and 87.1%, reduction, respectively), nadroparin (1000 or 2000 IU/kg, 36 and 60.7% reduction, respectively), and tinzaparin (3000 IU/kg, 41.3% reduction). All tested heparins also suppressed acid secretion in response to distention and histamine or bethanechol stimulation. Pretreatment with indomethacin did not abolish the gastric inhibitory action of nadroparin. After truncal vagotomy or atropine, nadroparin failed to inhibit acid secretion stimulated by histamine. Ganglionic blockade with guanethidine abolished the gastric inhibitory action of nadroparin or UFH. It is concluded that both UFH and LMWHs administered peripherally inhibit stimulated gastric acid secretion in the rat. This effect of heparins is determined by cholinergic and partly by adrenergic mechanisms.

### INTRODUCTION

The past few years have witnessed the introduction of several low molecular weight heparin (LMWH) preparations for the prevention and treatment of various thromboembolic disorders[1,2]. Unfractionated heparin (UFH) is a heterogeneous mixture of polysaccharide chains ranging in molecular weight from about 3000 to 30,000 daltons (Da). LMWHs are fragments of UFH produced by controlled enzymatic or chemical depolymerization processes that yield chains with a mean molecular weight of about 5000[3]. Their lower molecular weight and shorter polysaccharide chain length resulted in distinct pharmacokinetic properties. LMWHs exhibit less nonspecific binding to plasma proteins and endothelium, improved bioavailability, longer plasma half-life, and more predictable response[4].

Because the depolymerization process results in structural changes to UFH[5], LMWHs are also likely to exert different pharmacological actions, distinct from their anticoagulant properties[6,7,8].

The vast majority of patients who are receiving these heparin preparations are certainly at stress[9], which is a risk factor for the development of gastric erosions and or gastric bleeding[10,11]. In addition, acute gastroduodenal lesions were more commonly found when endoscopy was performed early after admission in patients with acute venous thromboembolism[12]. It therefore looked pertinent to examine the effect of different LMWHs on gastric acid secretion. Gastric acid is an important factor in the genesis of gastroduodenal mucosal injury and acid suppression with the histamine H2 antagonists or proton pump inhibitors is a fundamental in prophylaxis or treatment of stress ulceration[11,13].

Early work also suggested an inhibitory effect for heparin on gastric acid secretion[14,15]. The present study aimed to evaluate the effect of three already marketed LMWHs, namely enoxaparin, nadroparin, and tinzaparin, as compared to conventional UFH on stimulated gastric acid secretory responses in urethane-anesthetized rat.

### MATERIALS AND METHODS

#### Animals

Sprague-Dawley strain rats, of both sexes, 120–130 g of body weight, were used throughout the study and housed under standardized conditions for light and temperature. Rats were deprived of food for 18 h before the experiments, but allowed free access to tap water. The number of rats used in each experiment is presented in the text in parenthesis.

#### Drugs

The following drugs were used: heparin sodium (Nile Pharmaceutical Co., Cairo, ARE), enoxaparin sodium (Clexane, Rhone-Poulenc, France), nadroparin calcium (Fraxiparin, Sanofi/Cedex, France), tinzaparin sodium (Innohep, Leo Pharmaceutical Products, Ballerup, Denmark), histamine dihydrochloride, bethanechol (methacholine chloride), and urethane (Sigma, USA). Drugs were freshly diluted in saline before use to obtain the necessary doses. The doses of different heparins used in this study were selected based on the human doses used in therapy of deep-vein thrombosis[16,17,18] after conversion to that of rat according to Paget and Barnes[19] (Table 1).

**TABLE 1**
**Human Doses of UFH and LMWH Preparations[16,17,18] and Equivalent Doses for Rats**

| Drug | Dose (Human) | Dose (Rat) |
|---|---|---|
| Enoxaparin | 40 mg/person (4000 IU) daily; maximum, 180 mg/day (18,000 IU) | 3.6 mg/kg (360 IU); maximum, 16.2 mg/kg (1620 IU) |
| Nadroparin | 7500 IU daily; maximum, 17,100 IU/day | 675 IU/day; maximum, 1539 IU |
| Tinzaprin | 175 IU/kg; maximum, 18,000 IU/day | 1200 IU/kg; maximum, 1620 IU/kg |
| UFH | 25,000–40,000 IU/day | 2250–3600 IU/kg |

The doses for rats used were calculated according to Paget and Barnes conversion tables[19].

### Study Design

Acid secretion was measured in the acute fistula rat anesthetized with urethane (1.25 g/kg, i.p.) and maintained at 37°C by means of a heating lamp[20]. In brief, the abdomen was opened through a midline incision, the pylorus ligated, and 1-cm-diameter, double-lumen, plastic gastric cannula was inserted through an incision in the forestomach and exited the abdomen through the midline incision. At the beginning of the experiments, the stomach was flushed with physiological saline. A period of at least 30 min was allowed for stabilization of gastric acid secretion. Then, the stomach was distended by filling with 2 ml of saline and clamping the outlet of the gastric cannula, which was maintained for 2 h. The distention was carried out with or without the administration of different heparins under study. Drugs were administered by intraperitoneal route after dilution in saline to obtain the necessary doses in 0.2-ml volume. Conventional UFH (1000 or 2000 IU/kg, i.p., n = 6/group), nadroparin (1000 or 2000 IU/kg, i.p., n = 7/group), enoxaparin (180 or 360 IU/kg, i.p., n = 6/group), or tinzaparin (1000–3000 IU/kg, i.p., n = 6/group) was i.p given. The control group received i.p. saline (0.2 ml).

In other groups of rats, gastric acid secretion was measured under the same conditions of gastric distention in addition to histamine (5 mg/kg, i.p., n = 6/group) or cholinergic stimulation (bethanechol 1.5 mg/kg, i.p., n = 6/group). The effect of UFH (2000 or 4000 IU/kg, i.p., n = 7/group), nadroparin (2000 or 4000 IU/kg, i.p., n = 7/group), enoxaparin (360 or 540 IU/kg, i.p., n = 6/group), or tinzaparin (1000 or 3000 IU/kg, i.p., n = 6/group) administered simultaneously with the secretagogues was studied. The control group received i.p. saline (0.2 ml).

The effect of nadroparin was also studied in rats subjected to bilateral subdiaphragmatic vagotomy or treated with atropine (1 mg/kg, i.p.). The stomach was then distended with 2 ml saline and histamine (5 mg/kg) was i.p. given. Nadroparin (2000 IU/kg, 0.2 ml, i.p.) or saline (0.2 ml, i.p.) was administered in the atropine-treated or vagotomized rats (n = 6/group). Two hours later, gastric contents were collected for determination of gastric acid output. In a further set of experiments, the effect of nadroparin (1000 or 2000 IU/kg, 0.2 ml, i.p.) was determined in rats treated with indomethacin (20 mg/kg, 0.2 ml, i.p.) or guanethidine (8 mg/kg, i.p.) administered 30 min prior to surgery and intragastric saline (2 ml) (n = 7/group). We have also examined the effect of UFH (12,500 IU/kg, i.p.,) on gastric acid secretion in rats treated with guanethidine (n = 8/group).

Rats received s.c. saline 1 ml/h to correct for possible dehydration during the experiment. At the end of the experimental period, gastric contents were collected, $H^+$ output determined by titration with 0.01*N* NaOH to pH 7, and expressed as μEq/2 h.

### Statistics

Results are means ± SEM. Data of gastric acid output are expressed as μEq/2 h/100 g. Data are analyzed using two-tailed Student's *t* test (when there were only two groups) or one-way ANOVA and Duncan's multiple range comparison test. A probability value less than 0.05 was considered statistically significant. The number of rats used in experiments is presented in the text in parenthesis.

## RESULTS

### Acid Secretion Stimulated by Gastric Distention

The acid secretory response induced by distention was inhibited by 19% by administration of 2000 IU/kg of UFH (21.0 ± 1.1 vs. 26.3 ± 2.0 μEq/2 h, $p < 0.05$). Enoxaparin (180 and 360 IU/kg) inhibited the gastric acid response by 59.2 and 87.1%, respectively (11.1 ± 0.7 and 3.5 ± 0.2 vs. 27.2 ± 1.3 μEq/2 h, $p < 0.05$). Nadroparin at 1000 and 2000 IU/kg inhibited the acid response by 36.9 and 60.7% (18.92 ± 1.1 and

11.92 ± 0.8 vs. 30.0 ± 2.0 μEq/2 h, $p < 0.05$). Tinzaparin administration (1000 or 2000 IU/kg) was without significant effect on gastric acid secretion, but higher dose of 3000 IU/kg inhibited acid secretion by 41.3% (13.8 ± 0.6 vs. 23.45 ± 1.4 μEq/2 h, $p < 0.05$) (Table 2).

**TABLE 2**
**Inhibition of Gastric Acid Secretion Stimulated by Distention by UFH and LMWHs in Urethane-Anesthetized Rats**

| Treatment | Gastric Acid Secretion (μEq/2 h) |
|---|---|
| Control | 26.3 ± 2.0 |
| UFH 1000 IU/kg | 24.1 ± 1.6 |
| UFH 2000 IU/kg | 21.0 ± 1.1* |
| | |
| Control | 27.2 ± 1.3 |
| Enoxaparin 180 IU/kg | 11.1 ± 0.7* |
| Enoxaparin 360 IU/kg | 3.5 ± 0.2* |
| | |
| Control | 30.0 ± 2.0 |
| Nadroparin 1000 IU/kg | 18.92 ± 1.1* |
| Nadroparin 2000 IU/kg | 11.92 ± 0.8* |
| | |
| Control | 23.45 ± 1.4 |
| Tinzaparin 1000 IU/kg | 20.1 ± 1.6 |
| Tinzaparin 2000 IU/kg | 19.6 ± 1.2 |
| Tinzaparin 3000 IU/kg | 13.8 ± 0.6* |

Data represent mean ± SEM of 2-h collection of gastric acid secretion. The control group was treated with the vehicle (saline). *p < 0.05 vs. corresponding control group. One-way ANOVA and Duncan's multiple range comparison test. n = 6–7/group.

### Acid Secretion Stimulated by Gastric Distention and Histamine

The administration of histamine (5 mg/kg, i.p.) produced a marked increase in gastric acid secretion in the presence of gastric distention (Table 3). The total acid response obtained with histamine was 28.3% inhibited after the administration of 4000 IU/kg of UFH (133.3 ± 10.0 vs. 185.8 ± 11.8 μEq/2 h, $p < 0.05$). Enoxaparin given at 540 IU/kg together with histamine inhibited the gastric acid secretory response by 32.2% (123.7 ± 8.4 vs. 182.6 ± 12.1 μEq/2 h, $p < 0.05$). After nadroparin (2000 or 4000 IU/kg), the gastric acid response was inhibited by 40 and 57.9%, respectively (120 ± 9.6 and 84.2 ± 6.1 vs. 200.3 ± 16.0 μEq/2 h, $p < 0.05$). Tinzaparin administered at 1000 IU/kg was ineffective, but higher dose of 3000 IU/kg inhibited gastric acid by 32.8% (152.1 ± 12.3 vs. control value of 226.4 ± 16.8 μEq/2 h, $p < 0.05$).

**TABLE 3**
**Inhibition of Gastric Acid Secretion Stimulated by Distention and Histamine (5 mg/kg, i.p.) by UFH and LMWHs in Urethane-Anesthetized Rats**

| Treatment | Gastric Acid Secretion (μEq/2 h) |
|---|---|
| Control | 185.8 ± 11.8 |
| UFH 2000 IU/kg | 178.2 ± 13.9 |
| UFH 4000 IU/kg | 133.3 ± 10.0* |
| | |
| Control | 182.6 ± 12.1 |
| Enoxaparin 360 IU/kg | 155.5 ± 11.3 |
| Enoxaparin 540 IU/kg | 123.7 ± 3.4* |
| | |
| Control | 200.3 ± 16.0 |
| Nadroparin 2000 IU/kg | 120.0 ± 9.6* |
| Nadroparin 4000 IU/kg | 84.2 ± 6.1* |
| | |
| Control | 226.4 ± 16.8 |
| Tinzaparin 1000 IU/kg | 217.4 ± 15.1 |
| Tinzaparin 3000 IU/kg | 152.1 ± 12.3* |

Data represent mean ± SEM of 2-h collection of gastric acid secretion. The control group was treated with vehicle (saline). *$p < 0.05$ vs. corresponding control group. One-way ANOVA and Duncan's multiple range comparison test. n = 6–7/group.

### Acid Secretion Stimulated by Gastric Distention and Bethanechol

The total acid output in the presence of bethanechol (1.5 mg/kg, i.p.) after 2 h was inhibited by 49 and 51.5% by UFH (2000 and 4000 IU/kg, respectively) (68.6 ± 5.6 and 65.2 ± 5.0 vs. control value of 134.4 ± 11.0 μEq/2 h, $p < 0.05$). The gastric acid secretory response to bethanechol was also inhibited by nadroparin, enoxaparin, and tinzparin administered simultaneously with the secretagogue. At enoxaparin doses of 360 and 540 IU/kg, the gastric acid response was 27 and 39.6% inhibited (85.1 ± 6.6 and 70.4 ± 5.3 vs. 116.6 ± 8.3 μEq/2 h, $p < 0.05$). After nadroparin at 2000 or 4000 IU/kg, the gastric acid response was inhibited by 27.4 and 72.1%, respectively (96.8 ± 6.0 and 37.2 ± 3.0 vs. 133.3 ± 9.0 μEq/2 h, $p < 0.05$). Tinzaparin administration at 3000 IU/kg inhibited the gastric acid response by 48.5%, respectively (63.8 ± 5.1 vs.124 ± 8.6 μEq/2 h, $p < 0.05$) (Table 4).

### Effect of Subdiaphragmatic Vagotomy

After subdiaphragmatic vagotomy, nadroparin (2000 IU/kg) did not inhibit gastric acid secretion stimulated by histamine and gastric distention (33.6 ± 3.2 vs. control values of 39.3 ± 2.6 μEq/2 h) (Table 5).

**TABLE 4**
**Inhibition of Gastric Acid Secretion Stimulated by Distention and Bethanechol (1.5 mg/kg, i.p.) by by UFH and LMWHs in Urethane-Anesthetized Rats**

| Treatment | Gastric Acid Secretion (μEq/2 h) |
|---|---|
| Control | 134.4 ± 11.0 |
| UFH 2000 IU/kg | 68.6 ± 5.6* |
| UFH 4000 IU/kg | 65.2 ± 5.0* |
| | |
| Control | 116.6 ± 9.3 |
| Enoxaparin 360 IU/kg | 85.1 ± 6.6* |
| Enoxaparin 540 IU/kg | 70.4 ± 5.3* |
| | |
| Control | 133.3 ± 9.1 |
| Nadroparin 2000 IU/kg | 96.8 ± 6.0* |
| Nadroparin 4000 IU/kg | 37.2 ± 3.0* |
| | |
| Control | 124.0 ± 8.6 |
| Tinzaparin 1000 IU/kg | 108.0 ± 7.6 |
| Tinzaparin 3000 IU/kg | 63.8 ± 5.1* |

Data represent mean ± SEM of 2-h collection of gastric acid secretion. The control group was treated with vehicle (saline). *$p < 0.05$ vs. corresponding control group. One-way ANOVA and Duncan's multiple range comparison test. n = 6–7/group.

### Effect of Atropine

In rats treated with 1 mg/kg atropine, nadroparin (2000 IU/kg) did not decrease gastric acid secretion stimulated by histamine and gastric distention (84 ± 6.1 vs. control values of 93.2 ± 7.3 μEq/2 h) (Table 5).

### Effect of Indomethacin

The total acid output in rats pretreated with indomethacin was inhibited by 46.4% by nadroparin administration at 2000 IU/kg (12 ± 0.7 vs. 22.4 ± 1.8 μEq/2 h; $p < 0.001$) (Table 5).

### Effect of Guanethidine

In rats treated with 8 mg/kg guanethidine, nadroparin (1000 or 2000 IU/kg) did not decrease gastric acid secretion (20.2 ± 2.3, n = 6 and 18.6 ± 1.4 vs. control values of 16.3 ± 1.3 μEq/2 h). Only when high doses of heparin (12500 IU/kg) were administered to guanethidine-treated rats, 18.5% decrease in gastric acid was noted relative to the control group (26.0 ± 1.1 vs. 21.2 ± 1.6 μEq/2 h; $p < 0.05$) (Table 5).

**TABLE 5**
**Effect of Nadroparin on Distention Plus Histamine-Induced (5 mg/kg, i.p.) Stimulation of Gastric Acid Secretion after Subdiaphragmatic Vagotomy, Atropine (1 mg/kg, i.p.), Indomethacin (20 mg/kg, i.p.), or Guanethdine (8 mg/kg, i.p.) Treatment in Urethane-Anesthetized Rats**

| Treatment | Gastric Acid Secretion (μEq/2 h) |
|---|---|
| Vgotomy (control) | 39.3 ± 2.6 |
| + Nadroparin 2000 IU/kg | 33.6 ± 3.2$^{NS}$ |
| Atropine (control) | 93.2 ± 7.3 |
| + Nadroparin 2000 IU/kg | 84.0 ± 6.1$^{NS}$ |
| Indomethacin (control) | 22.4 ± 1.8 |
| + Nadroparin 2000 IU/kg | 12.0 ± 0.7* |
| Guanethdine (control) | 16.3 ± 1.3 |
| + Nadroparin 2000 IU/kg | 18.6 ± 1.4$^{NS}$ |

Data represent mean ± SEM of 2-h collection of gastric acid secretion. The control group was treated with vehicle (saline). n = 6/group. NS: not significant vs. corresponding control group. *$p < 0.001$ vs. indomethacin control group. Student's *t* test.

### DISCUSSION

The present study in urethane-anesthetized gastric fistula rats provided evidence that both UFH and three different LMWHs (nadroparin, enoxaparin, and tinzaparin) inhibit gastric acid secretion stimulated by gastric distention alone or gastric distention in addition to histamine or bethanechol administration. Findings from the present work also suggest that nadroparin, enoxaparin, and tinzaparin were more effective in inhibiting gastric acid secretion than the conventional UFH; with enoxaparin being more potent in this respect.

LMWHs have different methods of preparation, which result in variations in mean molecular weight (MW), distribution of MW, and pharmacokinetic and pharmacodynamic profiles. Unlike other LMWHs, tinzaparin is prepared by enzymatic hydrolysis with heparinase, while various chemical depolymerization methods are used for the synthesis of the rest of LMWHs. The mean MW of these compounds ranges from 3600 to 6500 Da. Each of the LMWHs exhibit distinct pharmacologic and biochemical profiles because of manufacturing differences[21,22]. Heparin has a short half-life in plasma ($t1/2 = 1.5$ h) and has variable and extensive binding to plasma proteins and cells. In comparison, LMWHs have superior pharmacologic and pharmacokinetic properties. This is because they do not bind to the endothelium and have a lower affinity for plasma proteins, resulting in better bioavailability, a substantially longer half-life, and a stable dose/response relationship when injected subcutaneously. For example, LMWHs are nearly three times as bioavailable as UFH when administered subcutaneously. This latter property is likely to account for the greater acid inhibitory effect of the LMWHs observed in present study. Pharmacokinetic differences also exist among the currently available LMWH preparations[1,3,4], which again is likely to be involved in the variability of their acid inhibitory effect.

In the present study, an attempt was made to investigate the mechanism by which the different heparin preparations inhibit gastric acid secretion. It is unlikely that the observed inhibition is due to prostaglandin release by these agents, since pretreatment with the cyclooxgenase inhibitor indomethacin did not affect the inhibition of acid by nadroparin. These antisecretory effects are mediated through vagal cholinergic pathways since they were abolished after subdiaphragmatic vagotomy or atropine treatment. Further, the antisecretory effect of nadroparin or UFH was not observed after peripheral sympathectomy induced by guanethidine pretreatment, suggesting that the mechanism of inhibition of acid secretion by heparins may involve modulation of the sympathetic nervous system. Distention of the stomach stimulates acid secretion by a vagal cholinergic mechanism[23,24]. The acid secretory response to distention in the rat also involves calcitonin gene-related peptide (CGRP)–containing neurons[25]. The response can be inhibited by vagotomy, anticholinergics, and H2-receptor antagonists[23,26,27] and is also subject to central modulation by sympathetic fibers[28,29]. The site of action of peripherally administered heparins in inhibiting gastric acid cannot be determined from the present experiments. Heparin and related molecules possess biological actions beyond their anticoagulant properties. It has been suggested that the relatively high plasma diamine oxidase activity observed following heparin bolus administration would lead to the rapid degradation of histamine and other diamine oxidase substrates present in the circulation[30]. Heparins might also act within the central nervous system to inhibit gastric acid secretion. Kondashevskaya et al.[6] have shown that rats treated with heparin exhibited increased concentrations of norepinephrine in the hypothalamus, homovanillic acid in the striatum, and serotonin in the small intestine.

Recent studies suggested that both UFH and LMWH given by intravenous or intragastric routes enhance gastric ulcer healing in rats. Increase in mucosal regeneration and proliferation, angiogenesis, mucus content, nitric acid, or prostaglandin release contribute to the effects of heparins[31,32,33]. Findings from the present work may provide additional explanation for the ulcer-healing properties of UFH and LMWHs observed in experimental conditions. Ulcers are more likely to heal in the absence of acid and the use of antacids, H2-receptor blockers, or proton pump inhibitors is a cornerstone in therapy of gastric or duodenal ulcers[34,35]. It has been shown that omeprazole completely reversed the indomethacin-induced decrease in the microvessel density. The effect has been attributed to decreased gastric acid–pepsin attack at the ulcer base, as well as the decreased degradation of acid-unstable fibroblast growth factors[36].

In summary, the present study indicates that both UFH and LMWHs administered peripherally inhibit stimulated gastric acid secretion in the rat. This effect is determined by cholinergic and partly by adrenergic mechanisms.

## References

1. Hirsh, J. and Raschke, R. (2004) Heparin and low-molecular-weight heparin: the Seventh ACCP Conference on Antithrombotic and Thrombolytic Therapy. *Chest* **126(Suppl 3),** 188S–203S.
2. Holzheimer, R.G. (2004) Low-molecular-weight heparin (LMWH) in the treatment of thrombosis. *Eur. J. Med. Res.* **9**, 225–239.
3. Weitz, J.I. (1997) Low-molecular-weight heparins. *N. Engl. J. Med.* **337**, 688–697.
4. Ageno, W. and Huisman, M.V. (2000) Low-molecular-weight heparins in the treatment of venous thromboembolism. *Curr. Control Trials Cardiovasc. Med.* **1,** 102–106.
5. Linhardt, R.J., Loganathan, D., al-Hakim, A., Wang, H.M., Walenga, J.M., Hoppensteadt, D., and Fareed, J. (1990) Oligosaccharide mapping of low molecular weight heparins: structure and activity differences. *J. Med. Chem.* **33,** 1639–1645.
6. Kondashevskaya, M.V., Kudrin, V.S., Klodt, P.M., Chepurnova, N.E., and Chepurnov, S.A. (2000) New aspects of heparin effects. *Bull. Exp. Biol. Med.* **30,** 1134–1137.
7. Salbach, P.B., Bruckmann, M., Turovets, O., Kreuzer, J., Kubler, W., and Walter-Sack, I. (2000) Heparin-mediated selective release of hepatocyte growth factor in humans. *Br. J. Clin. Pharmacol.* **50**, 221–226.
8. Abdel-Salam, O.M.E., Baiuomy, A.R., Ameen, A., and Hassan, N.S. (2005) A study of unfractionated and low molecular weight heparins in a model of cholestatic liver injury in the rat. *Pharmacol. Res.* **51**, 59–67.
9. Devlin, J.W. (2000) The role of low-molecular-weight heparin for DVT prophylaxis in acutely ill medical patients. *J. Inform. Pharmacother.* **3**, 300–308.
10. Cho, C.H., Koo, M.W., Garg, G.P., and Ogle, C.W. (1992) Stress-induced gastric ulceration: its aetiology and clinical implications. *Scand. J. Gastroenterol.* **27**, 257–262.
11. Yang, Y.X. and Lewis, J.D. (2003) Prevention and treatment of stress ulcers in critically ill patients. *Semin. Gastrointest. Dis.* **14**, 11–19.
12. Monreal, M., Boix, J., Romeu, J., Arias, A., and Pujol, M.A. (1991) Acute gastroduodenal lesions in patients with venous thromboembolism. Identification of patients at risk. *Chest* **100**, 488–492.
13. van Leerdam, M.E. and Rauws, E.A.J. (2001) The role of acid suppressants in upper gastrointestinal ulcer bleeding. *Best Pract. Res. Clin. Gastroenterol.* **15**, 463–475.
14. Lerner, H.J. and Thompson, J.C. (1963) Heparin suppression of gastric acid secretion. *Proc. Soc. Exp. Biol. Med.* **112**, 730–732.
15. Thompson, J.C., Lerner, H.J., Tramontana, J.A., and Miller, J.H. (1966) Range of action of heparin in suppressing canine gastric acid secretion. *Surg. Gynecol. Obstet.* **22**, 264–268.
16. Bates, S.M. and Ginsberg, J.S. (2004) Treatment of deep-vein thrombosis. *N. Engl. J. Med.* **351**, 268–277.
17. Hyers, T.M., Agnelli, G., Hull, R.D., Morris, T.A., Samama, M., Tapson, V., and Weg, J.G. (2001) Antithrombotic therapy for venous thromboembolic disease. *Chest* **119**,176–193.
18. Dahlman, T.C. (1993) Osteoporotic fractures and the recurrence of thromboembolism during pregnancy and the puerperium in 184 women undergoing thromboprophylaxis with heparin. *Am. J. Obstet. Gynecol*. **168**, 1265–1270.
19. Paget, G.E. and Barnes, J.M. (1964) Toxicity tests. In *Evaluation of Drug Activities Pharmacometics*. Laurence, D.R. and Bacharach, A.L., Eds. Academic Press, London. pp. 1–135.
20. Raybould, H.E. and Tache, Y. (1989) Capsaicin-sensitive vagal afferent fibres and stimulation of gastric acid secretion in anesthetized rats. *Eur. J. Pharmacol.* **167**, 237–243.
21. Fareed, J., Jeske, W., Hoppensteadt, D., Clarizio, R., and Walenga, J.M. (1996) Are the available low-molecular-weight heparin preparations the same? *Semin. Thromb. Hemost.* **22(Suppl 1),** 77–91.
22. Samama, M.M. and Gerotziafas, G.T. (2000) Comparative pharmacokinetics of LMWHs. *Semin. Thromb. Hemost.* **26(Suppl 1),** 31.
23. Hakanson, R., Hedenbro, J., Liedberg, G., Sundler, F., and Vallgren, S. (1980) Mechanisms of gastric acid secretion after pylorus and oesophagus ligation in the rat. *J. Physiol.* **305,** 139–149.
24. Schubert, M.L. and Makhlouf, G.M. (1993) Gastrin secretion induced by distention is mediated by gastric cholinergic and vasoactive intestinal peptide neurons in rats. *Gastroenterology* **104**, 834–839.
25. Mailliard, M.E. and Wolfe, M.M. (1989) Effect of antibodies to the neuropeptide GRP on distention-induced gastric acid secretion in the rat. *Regul. Pept.* **26**, 287–296.
26. Vallgren, S., El-Munshid, H.A., Hedenbro, J., Rehfeld, J.F., and Hakanson, R. (1983) Mechanism of gastric acid response to pylorus ligation: effects of nephrectomy. *Scand. J. Gastroenterol.* **18**, 491–496.
27. Noto, T., Nagasaki, M., and Endo, T. (1997) Role of vagus nerves and gastrin in the gastric phase of acid secretion in male anesthetized rats. *Am. J. Physiol.* **272,** G335–339.
28. Humphreys, G.A., Davison, J.S., and Veale, W.L. (1992) Hypothalamic neuropeptide Y inhibits gastric acid output in rat: role of the autonomic nervous system. *Am. J. Physiol.* **263,** G726–732.
29. Yokotani, K., Murakami, Y., Okuma, Y., and Osumi, Y. (1997) Centrally applied nitric oxide donors inhibit vagally evoked rat gastric acid secretion: involvement of sympathetic outflow. *Jpn. J. Pharmacol.* **74**, 337–340.
30. Klocker, J., Perkmann, R., Klein-Weigel, P., Morsdorf, G., Drasche, A., Klingler, A., Fraedrich, G., and Schwelberger, H.G. (2004) Continuous administration of heparin in patients with deep vein thrombosis can increase plasma levels of diamine oxidase. *Vascul. Pharmacol.* **40**, 293–300.
31. Li, Y., Mei, Q.B., and Cho, C.H. (1998) Healing effects of heparin on acetic acid-induced gastric ulcers in rats. *Chin. Med. J. (Engl.)* **111**, 12–16.
32. Li, Y., Wang, W.P., Wang, H.Y., and Cho, C.H. (2000) Intragastric administration of heparin enhances gastric ulcer healing through a nitric oxide-dependent mechanism in rats. *Eur. J. Pharmacol.* **399**, 205–214.
33. Li, Y., Shin, V.Y., Cheuk, C.Y., Liu, E.S., and Cho, C.H. (2001) A 3.0-kDa low molecular weight heparin promotes gastric ulcer healing in rats. *Aliment. Pharmacol. Ther.* **15,** 2009–2017.
34. Chan, F.K.L. and Sung, J.JY. (2001) Role of acid suppressants in prophylaxis of NSAID damage. *Best Pract. Res. Clin. Gastroenterol.* **15**, 433–445.
35. Sung, J.J. (2003) The role of acid suppression in the management and prevention of gastrointestinal hemorrhage associated with gastroduodenal ulcers. *Gastroenterol. Clin. North Am.* **32(Suppl 3),** S11–23.
36. Schmassmann, A. (1998) Mechanisms of ulcer healing and effects of nonsteroidal anti-inflammatory drugs. *Am. J. Med.* **104(3A),** 43S–51S.

### BIOSKETCH

**Omar M.E. Abdel Salam, M.B.B.Ch, M.Sc., Ph.D.,** is Associate Professor of Pharmacology in the Department of Pharmacology at the National Research Centre in Cairo, Egypt. Dr. Abdel Salam received his M.B.B.Ch in Medicine (1985) and M.Sc. in Internal Medicine (1991) from the Faculty of Medicine at Cairo University, and later received his Ph.D. in Medical Sciences (1997) from the Hungarian Academy of Sciences in Budapest. He is a member of the Egyptian Society of Pharmacology and the Egyptian Society of Toxicology. His research interests include: gastric acid secretion and its regulation; gastric mucosal injury; NSAIDs-induced gastric mucosal injury; the role of sensory nerves in gastric mucosal protection; the effect of capsaicin on experimental gastric mucosal injury; hepatic protection; inflammation, analgesia, modulation by different agents; the role of antidepressants and the serotonin reuptake inhibitors in inflammation and nociception.
